# Celecoxib and sulindac inhibit TGF-β1-induced epithelial-mesenchymal transition and suppress lung cancer migration and invasion via downregulation of sirtuin 1

**DOI:** 10.18632/oncotarget.11127

**Published:** 2016-08-09

**Authors:** Byong-Ki Cha, Young-Suk Kim, Ki-Eun Hwang, Kyung-Hwa Cho, Seon-Hee Oh, Byoung-Ryun Kim, Hong-Young Jun, Kwon-Ha Yoon, Eun-Taik Jeong, Hak-Ryul Kim

**Affiliations:** ^1^ Department of Thoracic and Cardiovascular Surgery, Chonbuk National University Medical School, Jeonbuk, Korea; ^2^ Departments of Internal Medicine, Institute of Wonkwang Medical Science, Wonkwang University, School of Medicine 344-2 shinyong-dong Iksan, Jeonbuk, Korea; ^3^ Department of Premedicine, School of Medicine, Chosun University, Gwangju, Korea; ^4^ Department of Obstetrics & Gynecology, Wonkwang University, School of Medicine, Iksan, Jeonbuk, Korea; ^5^ Imaging Science Research Center, Wonkwang University, School of Medicine, Iksan, Jeonbuk, Korea; ^6^ Departments of Radiology, Wonkwang University, School of Medicine, Iksan, Jeonbuk, Korea

**Keywords:** celecoxib, sulindac, EMT, SIRT1, lung cancer

## Abstract

The non-steroidal anti-inflammatory drugs (NSAIDs) celecoxib and sulindac have been reported to suppress lung cancer migration and invasion. The class III deacetylase sirtuin 1 (SIRT1) possesses both pro- and anticarcinogenic properties. However, its role in inhibition of lung cancer cell epithelial-mesenchymal transition (EMT) by NSAIDs is not clearly known. We attempted to investigate the potential use of NSAIDs as inhibitors of TGF-β1-induced EMT in A549 cells, and the underlying mechanisms of suppression of lung cancer migration and invasion by celecoxib and sulindac. We demonstrated that celecoxib and sulindac were effective in preventing TGF-β1-induced EMT, as indicated by upregulation of the epithelial marker, E-cadherin, and downregulation of mesenchymal markers and transcription factors. Moreover, celecoxib and sulindac could inhibit TGF-β1-enhanced migration and invasion of A549 cells. SIRT1 downregulation enhanced the reversal of TGF-β1-induced EMT by celecoxib or sulindac. In contrast, SIRT1 upregulation promoted TGF-β1-induced EMT. Taken together, these results indicate that celecoxib and sulindac can inhibit TGF-β1-induced EMT and suppress lung cancer cell migration and invasion via downregulation of SIRT1. Our findings implicate overexpressed SIRT1 as a potential therapeutic target to reverse TGF-β1-induced EMT and to prevent lung cancer cell migration and invasion.

## INTRODUCTION

Of all lung cancers, non-small cell lung cancer (NSCLC) constitutes approximately 80%, with only a small subset of patients achieving long-term survival. Most NSCLC patients show locally advanced, inoperable, or metastatic disease [1] and their prognosis is typically poor. In many cases, standard therapies such as chemotherapy and radiotherapy show limited effects. Thus, novel treatment strategies are needed to target this aggressive disease.

Epithelial cells generally lose their epithelial characteristics, such as cell-cell contact and cell polarity, and acquire a spindle-shaped migrating phenotype during EMT. The switch of E-cadherin to N-cadherin, which is the key event in EMT, renders single cells more motile and invasive [2–6]. Therefore, halting EMT may represent a novel cancer treatment strategy by inhibiting migration and invasion of cancer. EMT can be initiated by several signals, such as transforming growth factor β (TGF-β), epidermal growth factor (EGF), fibroblast growth factor, and hepatocyte growth factor [7, 8]. TGF-β is a multifunctional cytokine that induces EMT during wound healing, embryonic development, fibrotic disease, and cancer progression [9]. TGF-β is not only the major mediator of EMT, but also is markedly related to epithelial-mesenchymal interactions during lung morphogenesis [10].

Several clinical trials have been performed to investigate the benefits of non-steroidal anti-inflammatory drugs (NSAIDs) in not only combination treatment with standard chemotherapy [11–14], but also the chemoprevention of premalignant lesions [11]. However, these trials did not exhibit a convincing chemopreventive effect or additional therapeutic effects of NSAIDs alone on clinical outcomes. This suggests that the ideal applications of NSAIDs should be reevaluated. Additionally, further studies are required to evaluate diverse mechanisms involving the chemotherapeutic effects of NSAIDs.

Sirtuins belong to the class III histone deacetylase family that includes seven mammalian isoforms (SIRT1-7). They deacetylate lysine residues in histones and non-histone proteins, using NAD^+^ as a substrate [15, 16]. SIRT1, which is mainly located in the nucleus, regulates gene expression by changing the structure of chromatin and by modulating the activities of transcription factors [17, 18]. SIRT1 also acts as a redox sensor, allowing cells to cope with metabolic imbalance under nutrition- or oxygen-deficient conditions [19]. Although there is evidence for SIRT1 involving in various cell regulatory and physiological processes, the role of SIRT1 in regulating lung cancer EMT remains unclear. We examined the involvement of SIRT1 in TGF-β1-induced EMT during the migration and invasion of lung cancer cells in this study.

NSAIDs have been appeared to reverse EMT by restoring E-cadherin expression in subsets of lung, gastric, colon, and bladder cancer [20–23]. However, in NSCLC, the effect of NSAIDs on regulating E-cadherin expression and its mechanism in this process, have not been investigated. Moreover, the specific mechanisms by which NSAIDs inhibit lung cancer migration and invasion remain uncertain. In this study, we evaluated whether NSAIDs affect TGF-β1-induced EMT, a critical process involved in the migration and invasion of lung cancer, and proposed the relevance of these findings in lung cancer progression. Specifically, we aimed to re-evaluate the therapeutic potentials of celecoxib and sulindac for NSCLC treatment, and clarify the molecular basis of their effects. We show that celecoxib and sulindac inhibit TGF-β1-induced EMT and suppress lung cancer migration and invasion, and that this process involves a SIRT1-mediated signaling pathway.

## RESULTS

### Baseline expression of SIRT1 in NSCLC cells and involvement of SIRT1 in TGF-β-1-induced EMT

**Figure 1:**
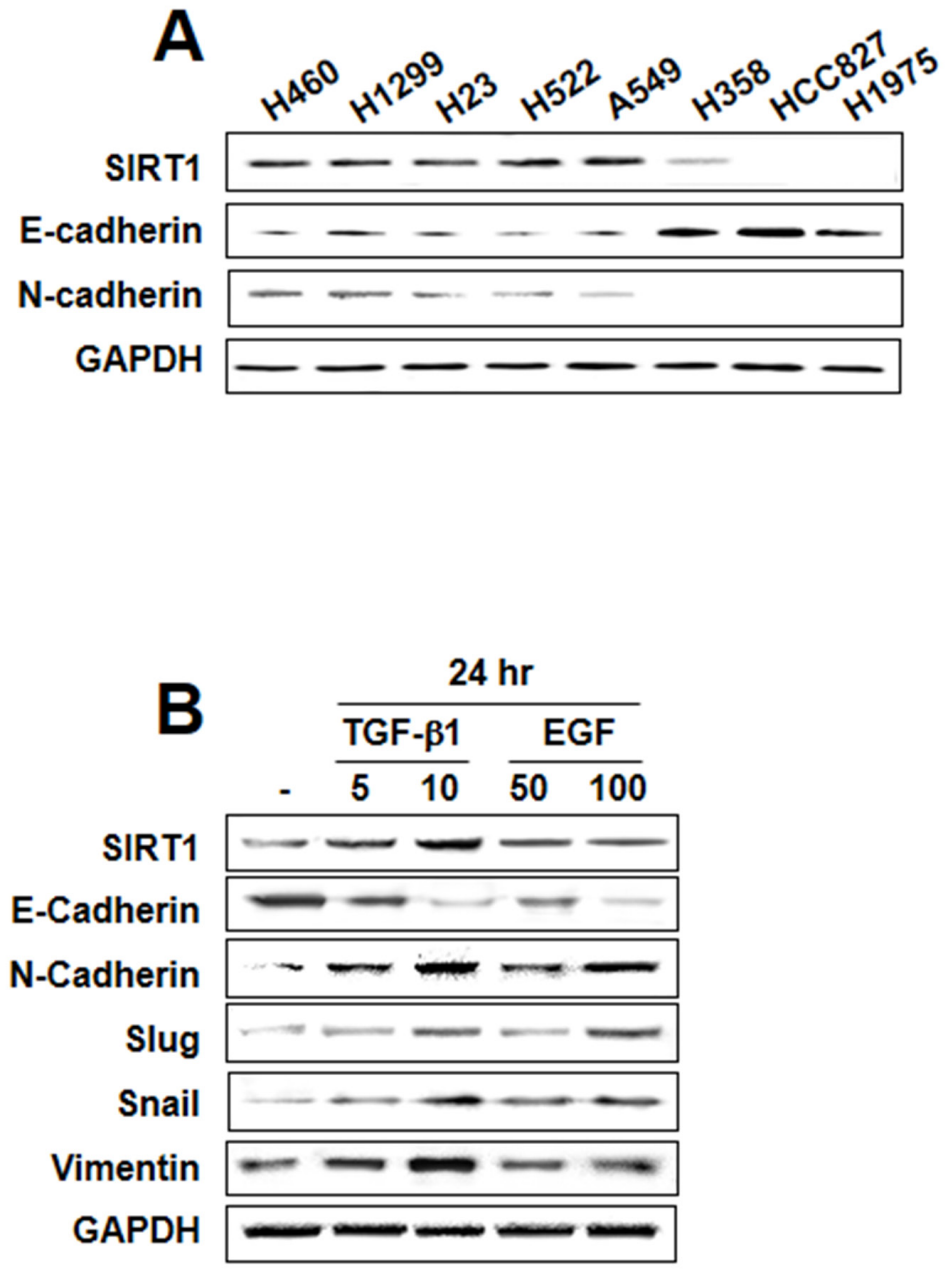
Transforming growth factor (TGF)-β1-induced sirtuin 1 (SIRT1) expression in lung cancer **A.** Endogenous expression of the epithelial-mesenchymal transition (EMT)-related proteins E-cadherin, N-cadherin, and SIRT1 was assessed in non-small cell lung cancer cell lines. **B.** A549 cells were treated with TGF-β1 (5 or 10 ng/mL) and epidermal growth factor (50 or 100 ng/mL) for 24 h. EMT hallmarks were examined using western blot analysis. Similar data were obtained from three independent experiments.

To determine the role of SIRT1 in regulating EMT in lung cancer, we initially screened eight human NSCLC cell lines for baseline expression of SIRT1 and cadherin proteins. Five cell lines (H460, H1299, H23, H522, and A549) showed moderate to high expression of SIRT1 at the protein level whereas in these cell lines, E-cadherin expression was inversely proportional to SIRT1 expression, and N-cadherin was constitutively expressed. In three other cell lines (H358, HCC827, and H1975) SIRT1 expression was weak or not detected; notably, a similar reciprocal relationship was also noticed between SIRT1 and E-cadherin expression in these cells (Figure 1A). These results indicate that SIRT1 may affect in regulating the EMT process. Studies have shown that cytokines, including TGF-β1 and EGF, induce morphological changes that are persistent with the acquisition of the EMT phenotype [24, 25]. Figure 1B shows that TGF-β1 or EGF treatment resulted in upregulation of transcription factors including Snail and Slug and mesenchymal markers including N-cadherin and vimentin, whereas E-cadherin was downregulated. Moreover, SIRT1 expression was increased in a concentration-dependent manner by treatment with TGF-β1, but not EGF. Therefore, we examined the role of SIRT1 on a TGF-β1-induced EMT model in A549 and H460 cells.

### TGF-β induces the expression of SIRT1 during EMT

**Figure 2:**
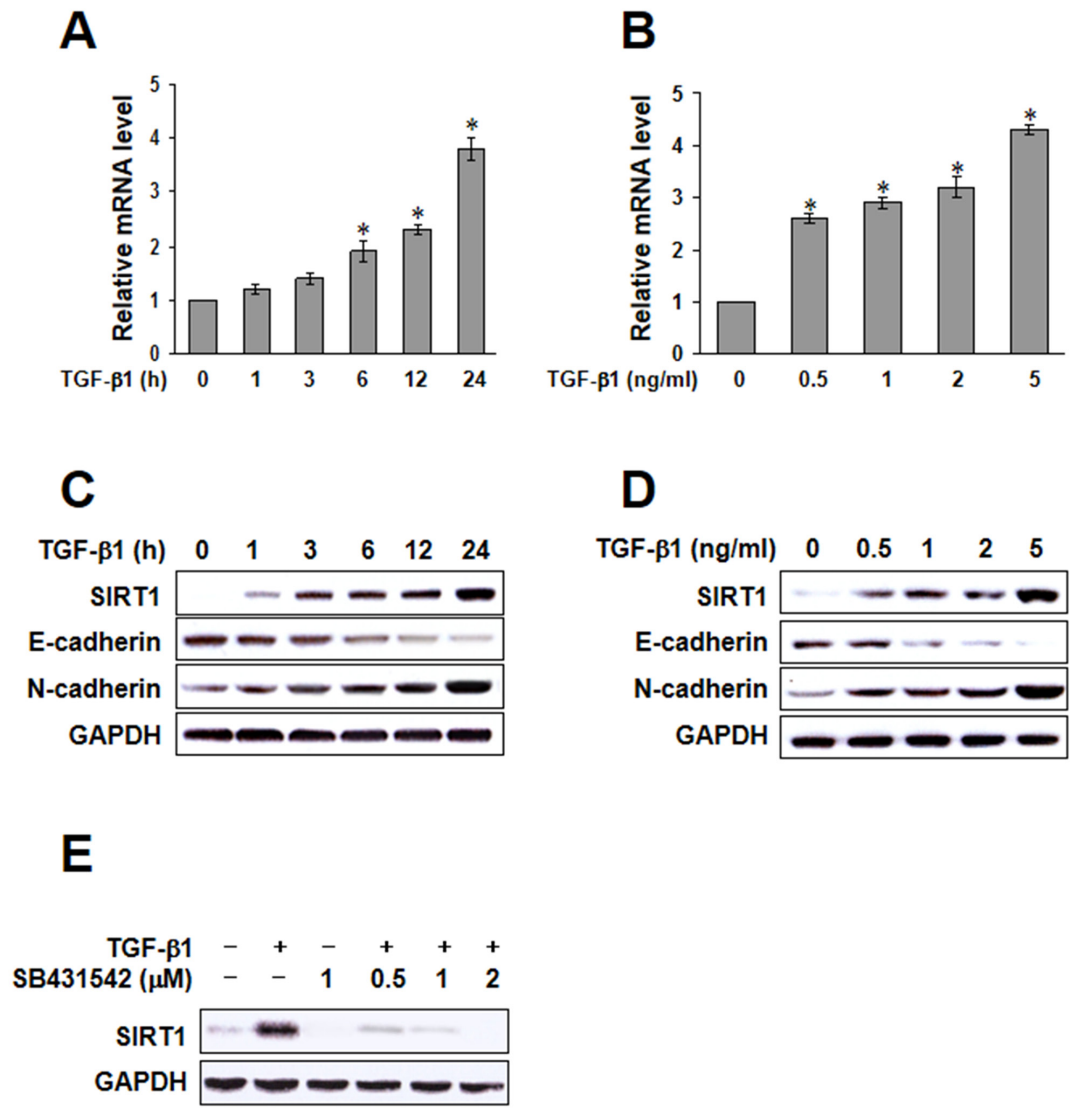
Transforming growth factor (TGF)-β1-induced sirtuin 1 (SIRT1) expression **A.** and **B.** A549 cells were treated with TGF-β1, and SIRT1 mRNA was quantified by real-time polymerase chain reaction in a time or dose dependent manner. The data represent the mean ± SD of three independent experiments. ^*^p < 0.05 compared to the control. **C** and **D.** A549 cells were treated with TGF-β1, and the expression of E-cadherin, N-cadherin, and SIRT1 was determined by immunoblotting. **E.** A549 cells were treated with 5 ng/mL TGF-β1, with or without SB431542 for 24h, and SIRT1 protein levels were examined by immunoblotting. Similar data were obtained from three independent experiments.

To improve the understanding of the mechanism of TGF-β1-induced EMT, we investigated whether SIRT1 is regulated by TGF-β in A549 cells. We verified that the mRNA level of SIRT1 was impressively increased after treatment with TGF-β1 in a time- and dose-dependent fashion (Figure 2A and 2B). We also confirmed by immunoblotting that TGF-β1 induced SIRT1 expression (Figure 2C and 2D). As shown in Figure 2E, upregulation of SIRT1 by TGF-β1 was rapid and sustained. This effect was blocked by SB431542, a selective inhibitor of TGF-β1, suggesting a role of SIRT1 in TGF-β1-induced EMT. Because EGF has also been shown to induce EMT-like morphological changes, we tested the effect of EGF on SIRT1 expression. However, SIRT1 expression was not increased in a time- or dose-dependent fashion by treatment with EGF. Moreover, SIRT1 was not blocked by AG1478, a selective inhibitor of EGF (Supplementary Figure S1A-S1C). Taken together, these results show that SIRT-1 confers epithelial cell plasticity, and that its level of expression affects the cellular response to EMT-inducing factors.

### Celecoxib and sulindac inhibit TGF-β1-induced EMT

**Figure 3:**
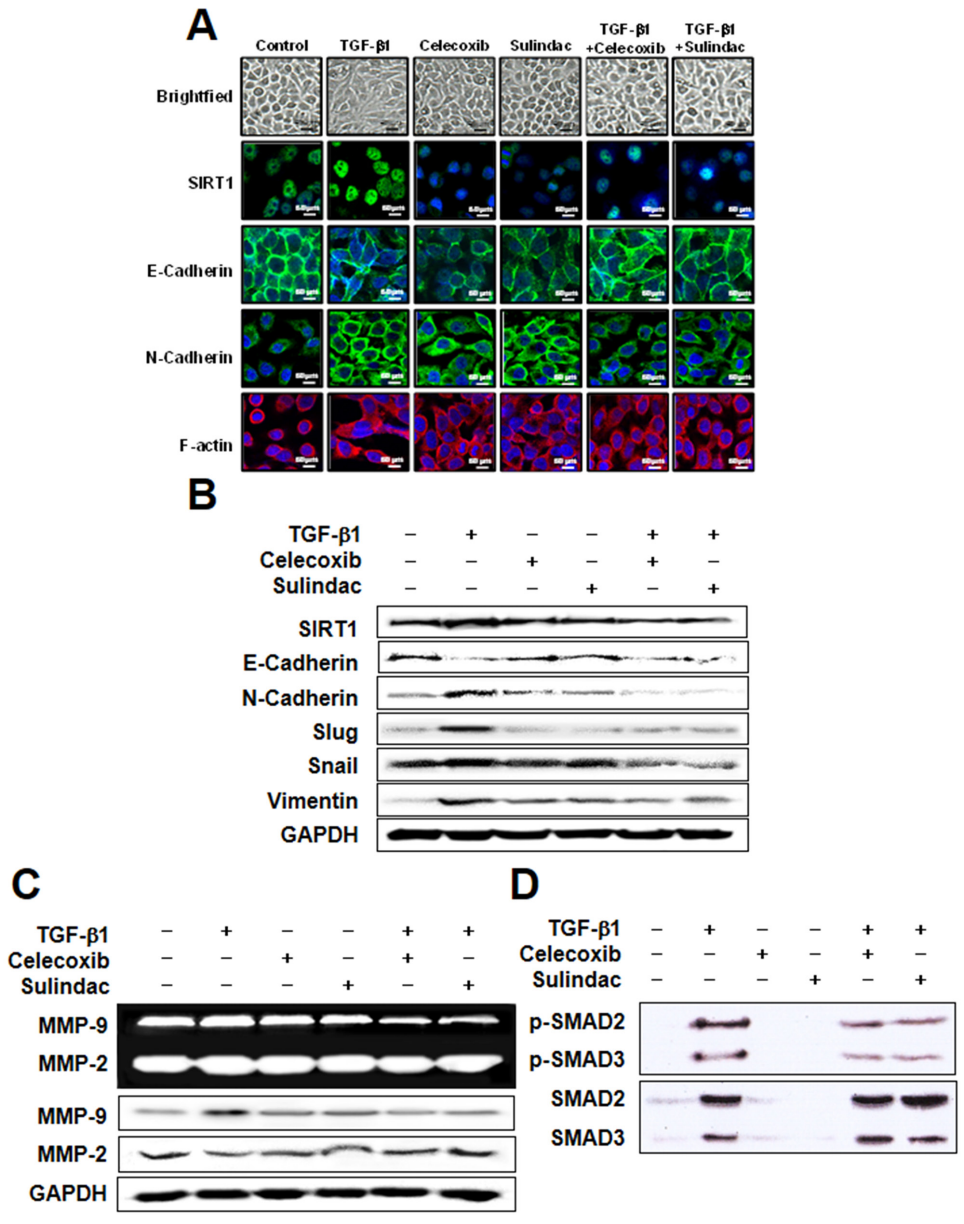
Celecoxib and sulindac inhibit transforming growth factor (TGF)-β1-induced epithelial-mesenchymal transition in A549 cells **A.** A549 cells were stimulated with 5 ng/mL TGF-β1 for 2 h and then incubated with 10 μM celecoxib or 500 μM sulindac for 48 h. Cell morphology was examined, and cells were fixed, permeabilized, and stained with anti-SIRT1, E-cadherin, and N-cadherin monoclonal antibody (green); and DAPI (blue). Cells were analyzed by confocal microscopy. All scale bars represent 60 μm. **B.** Western blot analysis using specific antibodies was performed to examine protein expression in whole cell lysates. Representative images from more than three independent experiments are shown. **C.** A549 cells were stimulated with 5 ng/mL TGF-β1 for 2 h and then incubated with 10 μM celecoxib or 500 μM sulindac for 48 h. The supernatants were analyzed by gelatin zymography, and cell lysates was subjected to 10% sodium dodecyl sulfate-polyacrylamide gel electrophoresis to measure the expression of MMP2 and MMP9. **D.** Cells were treated with 10 μM celecoxib or 500 μM sulindac in the absence or presence of 5 ng/mL TGF-β1. Cell lysates were then prepared and subjected to immunoblot analysis with antibodies to phosphorylated (p) or total forms of smad 2/3. Immunoblots are representative of at least three independent experiments.

The effect of celecoxib and sulindac on TGF-β1-induced EMT was next evaluated by investigating SIRT1 expression and cadherin switching using immunohistochemistry (Figure 3A). E-cadherin expression was declined in response to TGF-β1, whereas SIRT-1 and N-cadherin expression were increased. However, 10 μM celecoxib and 500 μM sulindac reversed SIRT-1 expression by TGF-β1 and inhibited the TGF-β1-induced cadherin switch. The inhibitory effect of celecoxib and sulindac on TGF-β1-induced EMT was also determined by visualizing the expression of EMT markers in A549 and H460 cells (Figure 3B, Supplementary Figure S2A). Consistent with Figure 3A, celecoxib and sulindac inhibited the decrease in E-cadherin expression and increase in vimentin expression that were induced by TGF-β1. Additionally, celecoxib and sulindac inhibited the TGF-β1-induced increase in expression of Snail and Slug. We next examined whether celecoxib and sulindac inhibit the activation of gelatinases, such as MMP2 and MMP9, and the expression of these proteins. Compared with controls, the expression of MMP-9 was upregulated by TGF-β1, an effect that was reversed by treatment with celecoxib and sulindac (Figure 3C, Supplementary Figure S2B). There was no change in expression of MMP-2 before or after treatment. To elucidate the anti-EMT mechanism of celecoxib and sulindac in detail, we tested the effect of these NSAIDs on TGF-β1-induced activation of Smad signaling. As shown in Figure 3D and Supplementary Figure S2C, TGF-β1 enhanced the phosphorylation of Smad2/3, but this effect was significantly decreased in the presence of celecoxib and sulindac. Collectively, these results indicate that celecoxib and sulindac antagonize the activation of MMP-9 and the phosphorylation of Smad2/3 promoted during TGF-β1-induced EMT in lung cancer.

### Celecoxib and sulindac inhibit TGF-β1-induced lung cancer cell migration

**Figure 4:**
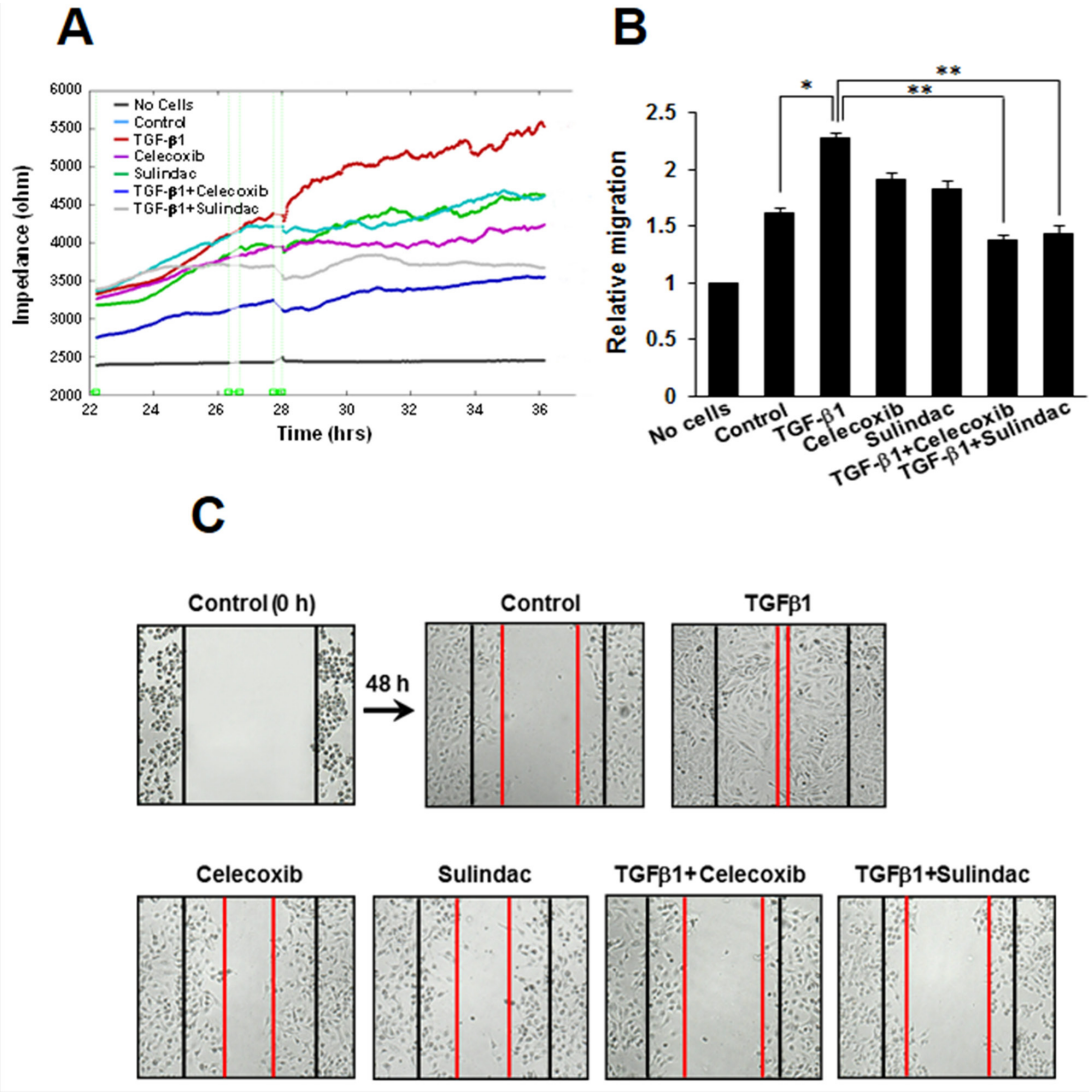
Effects of celecoxib and sulindac on transforming growth factor (TGF)-β1-induced A549 cell migration **A.** For the electric cell-substrate impedance sensing migration assay, A549 cells were stimulated with 5 ng/mL TGF-β1 for 2h and then incubated with 10 μM celecoxib or 500 μM sulindac for 48 h. Cell migration was then assessed by continuous resistance measurements for 40 h. **B.** The histogram represents the fold change in migration. The data represent the mean ± SD of three independent experiments. ^*^p < 0.05 compared with the control, ^**^p < 0.05 compared to the TGF-β1 group. **C.** Cell migration was also evaluated by wound healing assay. The confluent A549 monolayer was scratched with a pipette tip and washed to remove the debris. Fresh medium containing 0.5% serum was then added. Red lines indicate the cell edges at the T_0_ point. Representative pictures are shown.

To investigate the potential role of celecoxib and sulindac in inhibiting lung cancer cell migration, we assessed the effects of these NSAIDs on migration using an ECIS wound-healing assay and a scratch-migration assay. Using an ECIS-based quantitative real-time assay to follow the migration of A549 cells, we confirmed that cells treated with TGF-β1 showed increased resistance, whereas co-treatment with celecoxib or sulindac plus TGF-β1 resulted in decreased resistance. This shows that celecoxib and sulindac could inhibit TGF-β1-induced migration (Figure 4A and 4B). In a conventional scratch-migration assay (Figure 4C, Supplementary Figure S3), TGF-β1 increased the migration of lung cancer cells, whereas celecoxib and sulindac inhibited migration. These results indicate that celecoxib and sulindac are effective in the prevention of TGF-β1-induced lung cancer migration.

### Celecoxib and sulindac inhibit TGF-β1-induced A549 cell invasion

**Figure 5:**
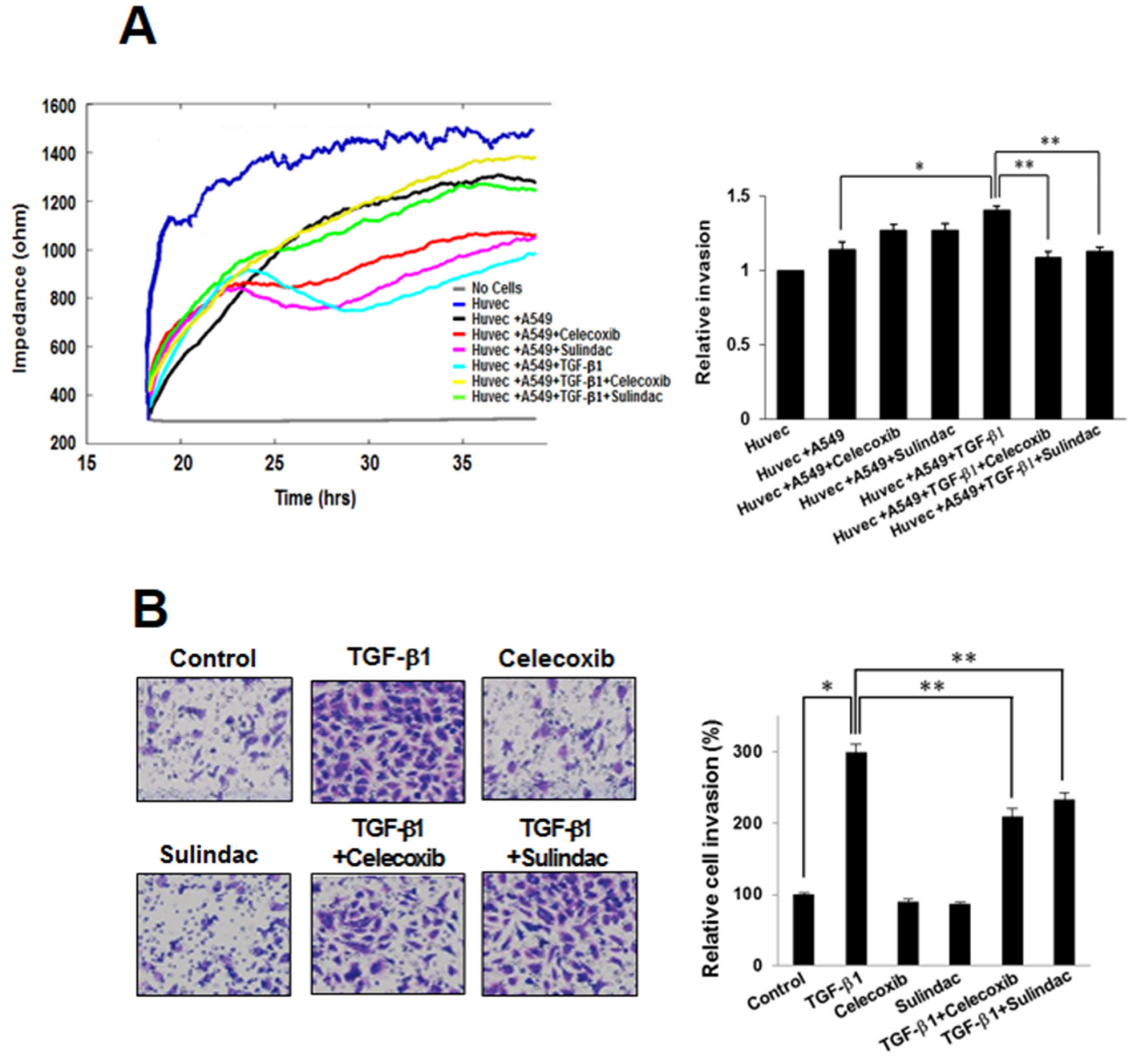
Effects of celecoxib and sulindac on transforming growth factor (TGF)-β1-induced A549 cell invasion **A.** For the electric cell-substrate impedance sensing invasion assay, resistance changes in the impedance at 4 kHz as confluent layers of HUVEC cells were challenged with A549 cells suspensions. The control curve of HUVEC cells received media without A549 cells. A549 cells were treated as above and changes in resistance were monitored for 40 h. The data represent the mean ± SD of three independent experiments. ^*^p < 0.05 compared with the control (HUVEC + A549), ^**^p < 0.05 compared to the HUVEC + A549 + TGF-β1 group. **B.** Effect of celecoxib and sulindac on A549 cell invasion in a 200× light microscope after crystal violet staining by matrigel invasion assay as described in Materials and Methods. Matrigel invasion of A549 cells counted in five random views. The data represent the mean ± SD of three independent experiments. ^*^p < 0.01 compared with the control, ^**^ *p* < 0.05 compared to the TGF-β1 group.

In order to confirm whether celecoxib and sulindac affect A549 cell invasion after stimulation by TGF-β1, we assessed the effects of these NSAIDs on invasion using an ECIS invasion assay and a matrigel invasion assay. In the ECIS-based invasion assay, established HUVEC cell layers were challenged with A549 cells. The decrease in resistance indicated that interactions occurred between the A549 cells and the HUVEC cells, and this led to the extravasation of A549 cells on the substratum. TGF-β1 induced a steep drop in resistance compared with untreated controls, demonstrating that TGF-β1 increased invasive potential. Celecoxib and sulindac inhibited the invasion of A549 cells, even in the presence of TGF-β1 (Figure 5A). We also assessed matrigel invasion assays to examine the effect of celecoxib and sulindac on the TGF-β1-induced invasion potential of lung cancer. TGF-β1 treatment increased invasion by A549 and H460 cells through matrigel in comparison to untreated cells, whereas celecoxib and sulindac inhibited invasion by lung cancer. Quantitative analysis indicates that nearly 80% of invasion was inhibited with celecoxib and sulindac (Figure 5B, Supplementary Figure S4). These results indicate that celecoxib and sulindac could effectively inhibit the TGF-β1-induced increase in invasion by lung cancer cells.

### Involvement of SIRT1 in TGF-β1-induced EMT inhibition by celecoxib and sulindac

**Figure 6:**
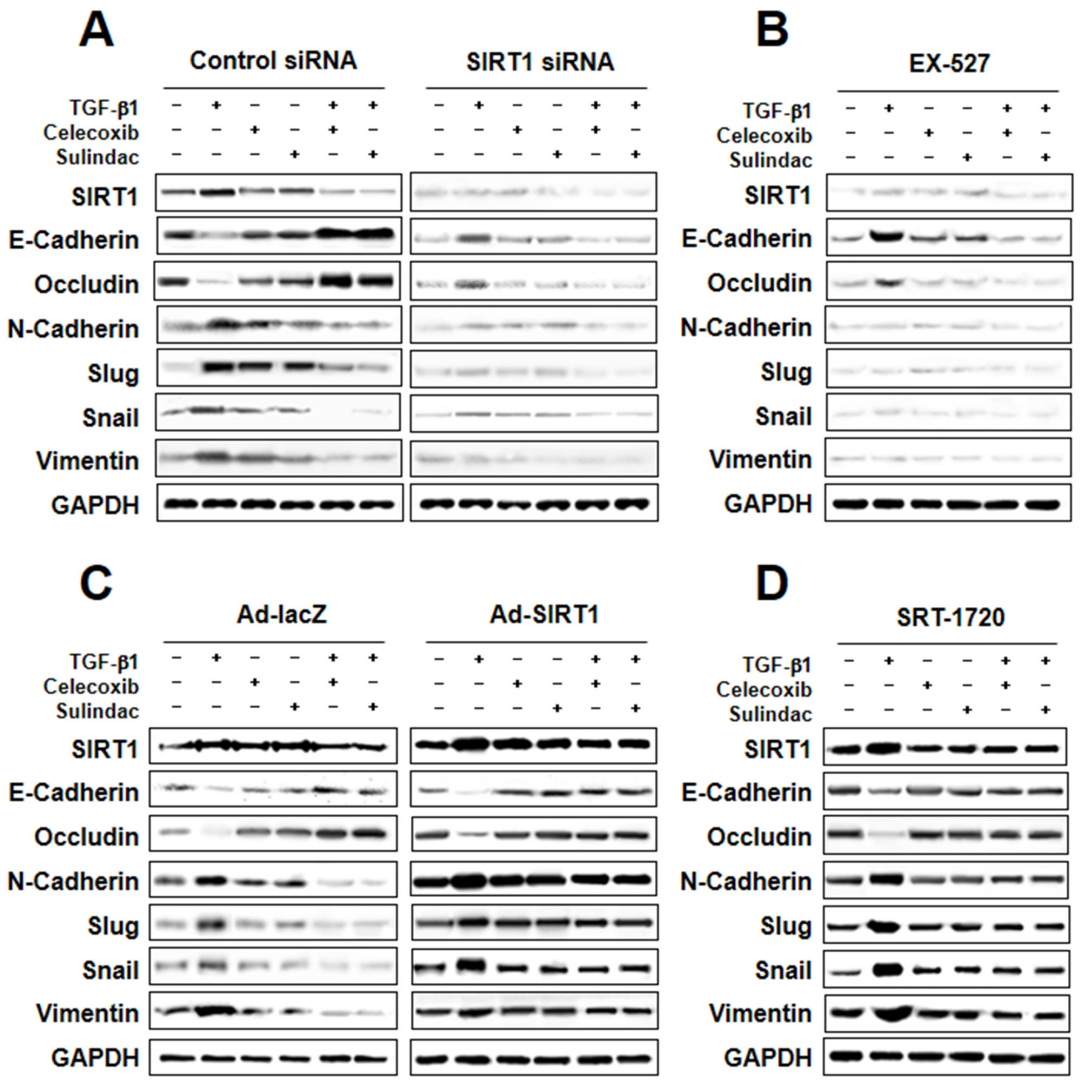
Involvement of sirtuin 1 (SIRT1) in transforming growth factor (TGF)-β1-induced epithelial-mesenchymal transition (EMT) inhibited by celecoxib and sulindac **A.** and **B.** Effect of SIRT1 inhibition on TGF-β1-induced EMT. Cells were transfected with SIRT1 siRNA or treated with the SIRT1 inhibitor EX-527, and then further incubated in the presence of celecoxib or sulindac for 24 h. The cell lysates of each group were prepared and probed for EMT hallmarks by western blot. **C** and **D.** Effect of SIRT1 activation on TGF-β1-induced EMT. Cells were transfected with Ad-lacZ or Ad-SIRT1, or treated with the SIRT1 activator SRT-1720, and then further incubated in the presence of celecoxib or sulindac for 24 h. The cell lysates were routinely prepared, and alterations in EMT hallmarks were determined by western blot.

To determine the role of SIRT1 in TGF-β1-induced EMT inhibition by celecoxib and sulindac, we first knocked down SIRT1 levels by introducing siRNA for SIRT1, or by treatment with the pharmacological SIRT1 inhibitor EX-527. Figure 6A shows that attenuation effect of TGF-β1-induced EMT resulted in treatment of SIRT1 siRNA in combination with celecoxib or sulindac. In the same way, when TGF-β1-induced A549 cells were treated EX-527 in combination with celecoxib or sulindac showed similar outcome (Figure 6B). These results indicate that SIRT1 knockdown or pharmacological inhibition can act synergistically with celecoxib and sulindac to inhibit TGF-β1-induced EMT.

We next examined whether SIRT1 upregulation could confer protection against TGF-β1-induced EMT inhibition by celecoxib and sulindac. We introduced SIRT1-expressing adenovirus into A549 cells, or treated cells with the SIRT1 activator SRT-1720. As shown in Figure 6C and 6D, both SIRT1 overexpression and SRT-1720 treatment markedly increased TGF-β1-induced EMT, even in the presence of celecoxib or sulindac.

To evaluate the role of COX-2 in mediating the effects of celecoxib and sulindac on EMT and SIRT1 regulation, we knocked down COX-2 levels by introducing an siRNA for COX-2, or by treatment with the pharmacological COX-2 inhibitor NS-398. As shown in Supplementary Figure S5A and S5B, inhibition of COX-2 by siRNA-mediated knockdown or treatment with NS-398 inhibited TGF-β1-induced EMT, and decreased the level of SIRT1. Taken together, these independent results indicate that SIRT1 and COX-2 are both involved in the inhibition of TGF-β1-induced EMT by celecoxib and sulindac.

### Downregulation of SIRT1 attenuates inhibition of TGF-β1-induced A549 cell migration and invasion by celecoxib

**Figure 7:**
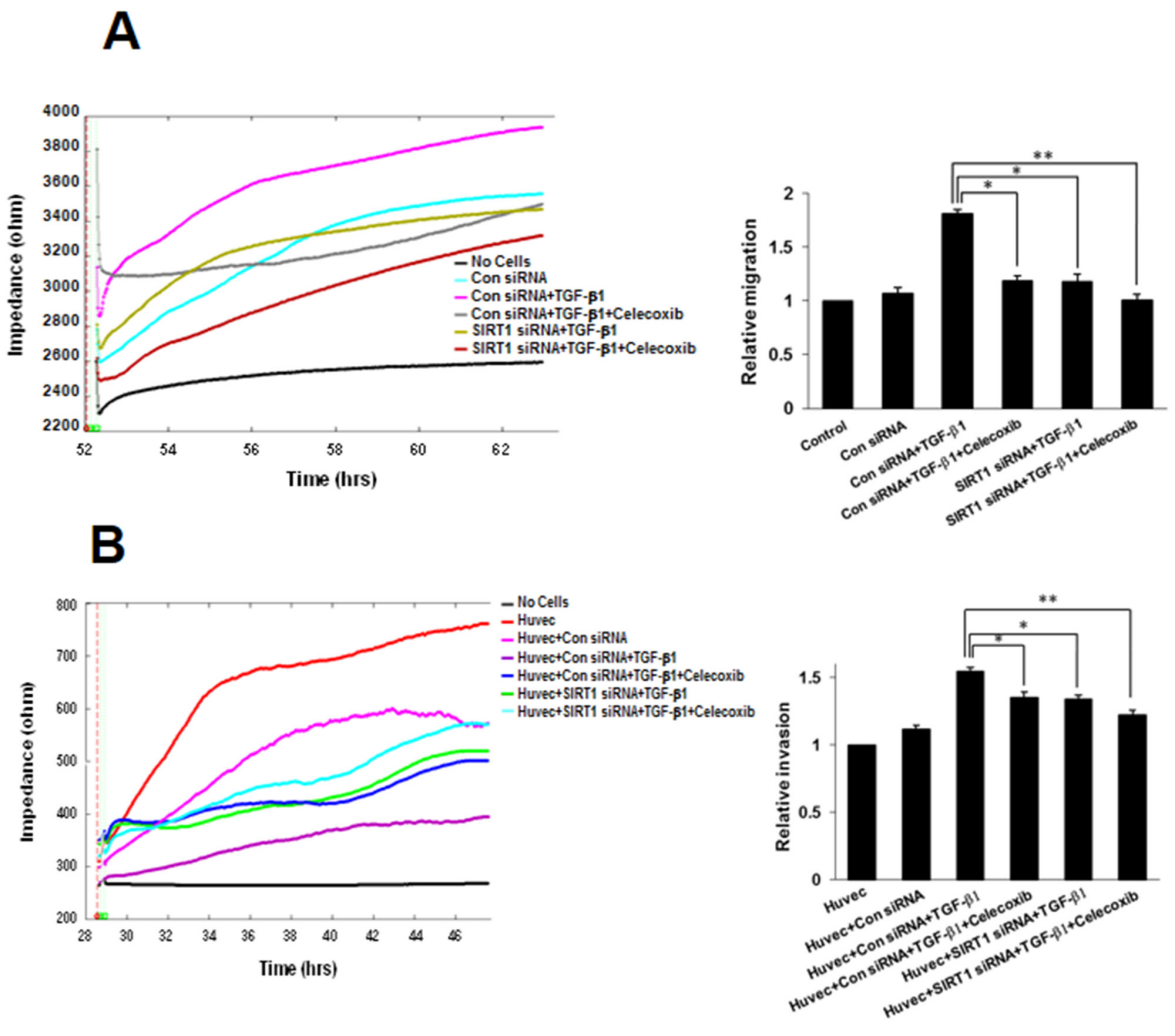
Effect of sirtuin 1 (SIRT1) deletion on transforming growth factor (TGF)-β1-induced A549 cell migration and invasion, inhibited by celecoxib **A.** Electric cell-substrate impedance sensing (ECIS) migration assay. A549 cells were transfected with siRNA specific for SIRT1. Control siRNA containing the same number of each nucleotide as the SIRT1 siRNA was used as the transfection control. Transfected cells were incubated in complete medium with TGF-β1 and/or 10 M celecoxib for 24 h. Cell migration was then assessed by continuous resistance measurements for 40 h. The data represent the mean ± SD of three independent experiments. ^*^p < 0.05 and ^**^ *p* < 0.01 compared with the control siRNA + TGF-β1. **B.** ECIS invasion assay. Resistance changes in the impedance at 4 kHz were measured as confluent layers of HUVEC cells were challenged with A549 cells suspensions. The control curve of HUVEC cells received media without A549 cells. A549 cells were treated as above and changes in resistance were monitored for 40 h. The histogram represents the fold change in migration or invasion. The data represent the mean ± SD of three independent experiments. ^*^p < 0.05 and ^**^ *p* < 0.01 compared with HUVEC + control siRNA + TGF-β1.

We next inspected the effects of SIRT1 on migration and invasion using an ECIS wound-healing assay and ECIS invasion assay, respectively. SIRT1 silencing resulted in marked attenuation of inhibition of TGF-β1-induced A549 cell migration by celecoxib (Figure 7A). Similarly, ECIS invasion assays also showed that the combination of SIRT1 siRNA and celecoxib synergistically decreased TGF-β1-induced A549 cell invasion compared with A549 cells treated with TGF-β1 alone (Figure 7B). These data indicate that the reduction of SIRT1 expression has an additive effect on the attenuation of lung cancer migration and invasion by celecoxib.

## DISCUSSION

In this study, we showed that the NSAIDs celecoxib and sulindac suppress lung cancer migration and invasion by inhibiting TGF-β1-induced EMT, and that NSAID-induced EMT inhibition in lung cancer may be attributed, at least partially, to a SIRT1-mediated pathway. This is the first study to show that increased expression of SIRT1 performs an important role in TGF-β1-induced EMT in lung cancer. Downregulation of SIRT1 significantly attenuates the TGF-β1-induced EMT-associated migration and invasion by lung cancer cells, indicating that SIRT1 enhances cancer cell motility during EMT, and therefore represents a novel target for cancer therapies.

The integrity of epithelial cells can be modified by altering the process of EMT. The plasticity of cancer cells may demonstrate the prometastatic effect of EMT during cancer progression. This plasticity may not only drive EMT of epithelial cells toward a mesenchymal state, but also induce basic changes in cell behavior and identity, including stemness, proliferation, migration, and invasion, all of which are involved in cancer progression [26, 27]. Recently, the importance of the tumor microenvironment in metastasis has been recognized. We confirmed that SIRT1 is induced during the early stages of tumor progression by TGF-β1 and is essential for the oncogenic functions of TGF-β1 in lung cancer. It is not clear whether SIRT1 overexpression alone is sufficient to induce EMT in lung cancer.

The anti-cancer effects of NSAIDs have been previously reported, including inhibition of VEGF expression by celecoxib [28], suppression of invasiveness by NS-398 [29, 30] and celecoxib [31], inhibition of proliferation by celecoxib, nimsulide, and meloxicam [31, 32], and induction of apoptosis by celecoxib [32] and sulindac [33]. Since EMT and enhanced cell migration are likely to be closely linked, NSAID-induced suppression of EMT may also contribute to attenuating the invasiveness of cancer cells. Considering the multifaceted targets of NSAIDs, several mechanisms have been considered to be related in their anti-cancer effects, and these mechanisms are thought to induce their effects in a cooperative manner.

SIRT1 has been shown to perform important roles in various processes such as stress responses, apoptosis, metabolism, and calorie restriction-linked longevity [34, 35]. SIRT1 regulates many genes by interacting with transcription factors [36, 37]. Many studies have indicated that SIRT1 functions as a positive regulator of EMT and, more specifically, metastatic growth of cancer cells [38]. SIRT1 has been suggested to play a role in epigenetic silencing of DNA-hypermethylated tumor suppressor genes in breast cancer cells [39, 40]. Recently, SIRT1 was found to be highly expressed in various cancers [41, 42], and high levels of SIRT1 expression were shown to be associated with a poor prognosis in lung cancer, breast cancer, B-cell lymphoma, and gastric carcinomas [43–45]. In prostate cancer, SIRT1 enhances cell migration and metastasis by cooperating with ZEB1 to suppress E-cadherin transcription. The SIRT1 activator compound 1720 showed that lung metastasis was increased by implanted breast cancer cells, suggesting that SIRT1 could function, at least under certain circumstances, as a tumor-promoting factor [46]. In contrast, several reports have described SIRT1 as a tumor suppressor that safeguards the organism from oncogenic stress. A recent report demonstrated that enhanced SIRT1 expression inhibited intestinal tumor formation in a β-catenin-dependent mouse model [47]. In lung cancer, downregulation of SIRT1 by hypoxia in a SUMOylation-dependent fashion facilitated EMT and resulted in cancer metastasis [48]. These results indicate that the effects of SIRT1 vary in different tumor models and may be conditional on the presence of proper downstream targets. Therefore, it is likely that the cellular characteristics and/or microenvironment may alter the impact of SIRT1 on EMT.

It is also very important to consider whether the concentrations of celecoxib and sulindac used in this study are clinically relevant and achievable. Davis et al. [49] reported that when celecoxib was administered to human subjects orally at a dose of 800 mg, the serum concentration reached up to 8 M. Furthermore, similar concentrations of celecoxib and sulindac that were used in the present study have been applied in other *in vitro* experiments [50, 51]. As the actual clinically relevant celecoxib and sulindac concentrations in the tissue are currently unclear, it is difficult to directly correlate the celecoxib and sulindac concentrations used *in vitro* to those that are clinically achievable. Nevertheless, it is possible that the *in vitro* mechanism of action of celecoxib and sulindac as described in this work is different from that occurring *in vivo*.

In this study, we showed that SIRT1 is a pivotal positive regulator of TGF-β-induced EMT and lung cancer migration, and invasion by NSAIDs. Together, celecoxib and sulindac may be a promising anticancer therapy for lung cancer treatment. Additional studies are needed to examine other molecular targets and mechanisms of EMT in lung cancer and to demonstrate the efficacy and safety of celecoxib and sulindac in lung cancer treatment.

## MATERIALS AND METHODS

### Materials

Roswell Park Memorial Institute medium 1640 (RPMI 1640), fetal bovine serum (FBS), celecoxib, sulindac, and antibiotics were obtained from GIBCO BRL Co. (Grand Island, NY, USA). SRT-1720 and EX-527 were purchased from Sigma-Aldrich Chemical Co. (St. Louis, MO, USA). Recombinant human TGF-β1 and EGF were purchased from R&D Systems (Abingdon, UK). Affinity purified monoclonal antibodies against mouse SIRT1 antibodies were obtained from Abcam (Cambridge, UK). Antibodies (Abs) against E-cadherin, N-cadherin, Slug, Snail, Vimentin, MMP2, MMP9, phospho-SMAD2/3, and SMAD2/3 were purchased from Cell Signaling Technology (Beverly, MA, USA). Anti-rabbit IgG-conjugated horseradish peroxidase (HRP) antibodies and enhanced chemiluminescence (ECL) kits were purchased from Amersham Pharmacia Biotech (Buckinghamshire, UK).

### Cell culture

Lung cancer cell lines H460, H1299, H23, H522, A549, H358, HCC827, and H1975 were purchased from the American Type Culture Collection (ATCC). These cell lines were grown in RPMI 1640 containing 100 units/mL penicillin, 0.1 mg/mL streptomycin, and 10% FBS. All cell lines used in the study were authenticated by the ATCC and KCLB using STR-PCR analysis. Cells were incubated in a humidified atmosphere of 5% CO_2_ in air at 37°C and maintained in log phase growth.

### Real-time polymerase chain reaction (PCR) analysis

Real-time PCR was performed on complementary DNA (cDNA) samples using the SYBR Green system (Bio Rad, Richmond, CA). Primers used were SIRT1, sense 5′- TCAGTGTCATGGTTCCTTTGC-3′ and anti-sense 5′-TCCACCACCCTGTTGCTGTA-3′. The following general real-time PCR protocol was employed: denaturation for 10 min at 95°C, 40 cycles of a four segmented amplification and quantification program, a melting step by slow heating from 60°C to 99°C with a rate of 0.1°C/sec and continuous fluorescence measurement, and a final cooling step, down to 40°C. Crossing point values were acquired by using the second derivative maximum method of the LightCycler software 3.3 (Roche, Burlington, NC). Real-time PCR efficiencies were acquired by amplification of a standardized dilution series, and slopes were determined using LightCycler software.

### Western blotting

Cells were harvested and lysed using a radioimmunoprecipitation assay buffer (50 mM Tris-Cl [pH 7.4], 1% NP40, 150 mM NaCl, 1 mM EDTA, 1 mM phenylmethylsulfonyl fluoride, 1 μg/mL aprotinin, 1 μg/mL leupeptin, and 1 mM Na_3_VO_4_). After centrifugation at 12,000× *g* for 30 min, supernatant was collected, and the protein concentration was determined by the Bradford method (Bio-Rad Protein Assay). Equal amounts of protein were separated using 12% sodium dodecyl sulfate-polyacrylamide gel electrophoresis (SDS-PAGE) under reducing conditions and subsequently transferred to nitrocellulose membranes. The membranes were blocked with 5% skim milk in TBS-T (25 mM Tris [pH 7.6], 138 mM NaCl, and 0.05% Tween-20) for 1 h and probed with primary antibodies (at 1:1000–1:5000 dilutions). After washing, the membranes were further incubated with a HRP-conjugated secondary antibody (at 1:2000–1:10,000 dilutions). Immunoreactive signals were detected using an ECL detection system.

### Immunofluorescence

Cells grown on chamber slides were washed with PBS for 15 min (total), fixed in 4% paraformaldehyde for 30 min at room temperature (RT), and permeabilized with 0.1% TritonX-100 at RT for 10 min. After blocking with goat serum for 2 h at RT, cells were incubated with antibodies against SIRT1, E-cadherin, N-cadherin, and F-actin (1:100 dilution) at 4°C overnight. Dishes were washed three times with PBS and incubated with Alexa-Fluor-488- or Alexa-Fluor-594-conjugated secondary antibodies (1:1000 dilution) for 1 h at RT. Nuclei were stained with DAPI (10 mg/mL) for 10 min. Samples were examined by confocal laser scanning microscopy (FV1000+IX2, Olympus America Inc, PA, USA) to analyze the expression of SIRT1, E-cadherin, N-cadherin, and F-actin.

### Gelatin zymography

To analyze MMP-2 and MMP-9 activity, we incubated A549 cells (1 × 10^5^ cells/well) in a 24-well plate for 24 h. After serum starvation for 24 h, the supernatant was collected after treated with celecoxib or sulindac in the absence or presence of TGF-β1and subjected to SDS-PAGE in 10% polyacrylamide gels with 1 mg/mL gelatin. After electrophoresis, gels were incubated in 2.5% Triton X-100 (1 h, 37°C) followed by overnight incubation in 50 mM Tris-HCl (pH 7.8), 5 mM CaCl_2_, 0.02% NaN_3_, 0.02% Brij gels, and stained with 2.5% Coomassie Blue R-250 (Bio-Rad) for 45 min, followed by destaining in deionized water with 10% acetic acid and 20% methanol. Gels were scanned and density analyses of the bands was performed using Photoshop CS4.0 (Alphalmager 2000, Alpha Innotech, San Leandro, CA).

### Electric cell-substrate impedance sensing (ECIS) wound-healing assay

Wound-healing assays were performed using ECIS (Applied BioPhysics, Troy, NY, USA) technology, following our previously established protocol [52]. For wound-healing assays, confluent A549 cells monolayers cultured on ECIS plates were submitted to an elevated voltage pulse of 60 kHz frequency, 3.5 V amplitude, and 30 s duration, leading to death and detachment of cells present on the small active electrode, resulting in a wound normally healed by cells surrounding the small active electrode that have not been submitted to the elevated voltage pulse. Wound healing was then assessed by continuous resistance measurements for 24 h.

### Scratch-migration assay

A549 cells were cultured in 6-well dishes (seeding density 1 × 10^6^ cells/well). Confluent cell monolayers were disrupted by standardized wound scratching using a sterile 200 μl pipette tip and incubated in culture medium with 1% FBS, with or without 5 ng/ml TGF-β1, 10 M celecoxib, 500 M sulindac, TGF-β1 plus celecoxib, or TGF-β1 plus sulindac for 48 h. Migration of cells into the bare area and recovering of the monolayer was evaluated every 12 h for 48 h using a phase contrast microscope, and was digitally photographed (Nikon Diaphot 300; Nikon, Tokyo, Japan).

### ECIS invasion assay

Electrode arrays were obtained from Applied BioPhysics (Troy, NY, USA), and ECIS invasion assays were performed as described previously [53]. ECIS array wells were precoated with a solution of 200 μg/ml gelatin in 0.15 mol/l NaCl. After 15 min of incubation to allow the gelatin to adsorb, the gelatin solution was aspirated, and the electrode-containing wells were rinsed twice with PBS. They were then partially filled with 200 μL Human Umbilical Vein Endothelial Cells (HUVEC) medium and allowed to equilibrate for 15 to 60 min in a humidified CO_2_ incubator. Approximately 1 × 10^5^ HUVECs were added to each well in 200 μL HUVEC medium. The attachment and spreading of cells into the ECIS wells was followed by impedance measurements using ECIS. HUVECs were challenged with monodispersed cell suspensions of A549 cells (20 × 10^5^/ml) in 50 μL fresh HUVEC medium. Triplicate wells were used for each treatment. Cells were treated with 1% FBS, with or without TGF-β1, celecoxib, sulindac, TGF-β1 plus celecoxib, or TGF-β1 plus sulindac. The impedance of the challenged endothelial cell layer was monitored via ECIS for the next 12 to 40 h.

### Matrigel invasion assay

Cell invasion assay kits (Chemicon International, Temecula, CA) were used to detect cell invasion according to the manufacturer's protocol. Cells were resuspended in culture media and incubated in a chemoinvasion chamber. A549 cells were seeded at a density of 2 × 10^4^ per insert and cultured for 12 h. Next, cells were placed in wells containing the same medium plus TGF-β1 (5 ng/ml), with or without celecoxib or sulindac. After 48 h, non-invading cells were removed with cotton swabs. The invasive capability of cells was measured as recommended by the manufacturer. Photomicrographs of the invasive cells were taken in five predetermined fields (magnification 200×) and quantification of stained cells was performed by dissolving cells in 10% acetic acid and measuring the optical density at 540 nm.

### Gene silencing

Pooled small interfering RNA (siRNA) oligonucleotides against SIRT1 were purchased from Cell Signaling Technology. Twenty-four hours after seeding, cells were transfected with 100 nM pooled oligonucleotide mixture using Lipofectamine 2000 (Invitrogen) according to the manufacturer's protocol. Twenty-four hours after transfection, media were removed and cells were treated with the indicated drugs. Gene silencing efficacy by siRNA was assessed by western blot analysis.

### Preparation of the recombinant adenovirus

To prepare SIRT1-expressing adenovirus, human SIRT1 cDNA was cloned into the KpnI and XhoI sites of pENTR 2B (Invitrogen), and the SIRT1 cDNA insert was transferred to the pAd/CMV/V5-DEST vector by the Gateway system using LR Clonase (Invitrogen). The plasmids were linearized with PacI (Promega, Madison, WI) and transfected into A549 cells using Lipofectamine 2000 (Invitrogen). As a control, the pAd/CMV/V5-GW/lacZ vector (Invitrogen) was used to produce lacZ-bearing adenovirus.

### Statistical analysis

Each experiment was performed at least three times, and all values were expressed as the mean ± SD of triplicate samples. Student's *t*-test was used to determine statistical significance. Values of *p* < 0.05 were considered statistically significant.

## SUPPLEMENTARY MATERIALS FIGURES



## References

[1] Jung KW, Won YJ, Kong HJ, Oh CM, Cho H, Lee DH, Lee KH (2015). Cancer statics in Korea: incidence, mortality, survival, and prevalence in 2012. Cancer Research and Treatment.

[2] Xu J, Lamouille S, Derynck R (2009). TGF-beta-induced epithelial to mesenchymal transition. Cell Research.

[3] Lee CM, Park JW, Cho WK, Zhou Y, Han B, Yoon PO, Chae J, Elias JA, Lee CG (2014). Modifiers of TGF-β1 effector function as novel therapeutic targets of pulmonary fibrosis. The Korean Journal of Internal Medicine.

[4] Cavellaro U, Christofori G (2004). Cell adhesion and signalling by cadherins and Ig-CAMs in cancer. Nature reviews. Cancer.

[5] Deckers M, van Dinther M, Buijs J, Que I, Löwik C, van der Pluijm G, ten Dijke P (2006). The tumor suppressor Smad4 is required for transforming growth factor beta-induced epithelial to mesenchymal transition and bone metastasis of breast cancer cells. Cancer Research.

[6] Miyazono K (2009). Transforming growth factor-beta signaling in epithelial-mesenchymal transition and progression of cancer. Proceedings of the Japan Academy. Series B, Physical and Biological Sciences.

[7] Lee JM, Dedhar S, Kalluri R, Thompson EW (2006). The epithelial-mesenchymal transition: new insights in signaling, development, and disease. The Journal of Cell Biology.

[8] Liu LC, Tsao TC, Hsu SR, Wang HC, Tsai TC, Kao JY, Way TD (2012). EGCG inhibits transforming growth factor- β-mediated epithelial-to-mesenchymal transition via the inhibition of Smad2 and Erk1/2 signaling pathways in nonsmall cell lung cancer cells. Journal of Agricultural and Food Chemistry.

[9] Yang J, Weinberg RA (2008). Epithelial-mesenchymal transition: at the crossroads of development and tumor metastasis. Developmental Cell.

[10] Cardoso WV, Lu J (2006). Regulation of early lung morphogenesis: questions, facts and controversies. Development.

[11] Giardiello FM, Spannhake EW, DuBois RN, Hylind LM, Robinson CR, Hubbard WC, Hamilton SR, Yang VW (1998). Prostaglandin levels in human colorectal mucosa: effects sulindac in patients with familial adenomatous polyposis. Digestive Diseases and Sciences.

[12] Edelman MJ, Watson D, Wang X, Morrison C, Kratzke RA, Jewell S, Hodgson L, Mauer AM, Gajra A, Masters GA, Bedor M, Vokes EE, Green MJ (2008). Eicosanoid modulation in advanced lung cancer: cyclooxygenase-2 expression is a positive predictive factor for celective + chemotherapy-Cancer and Leukemia Group B Trial 20203. Journal of Clinical Oncology.

[13] Mutter R, Lu B, Carbone DP, Csiki I, Moretti L, Johnson DH, Morrow JD, Sandler AB, Shyr Y, Ye F, Choy H (2009). A phase II study of celecoxib in combination with paclitaxel, carboplatin, and radiotherapy for patients with inoperable stage IIIA/B non-small cell lung cancer. Clinical Cancer Research.

[14] Fujii R, Imanishi Y, Shibata K, Sakai N, Sakamoto K, Shigetomi S, Habu N, Otsuka K, Sato Y, Watanabe Y, Ozawa H, Tomita T, Kameyama K, Fujii M, Ogawa K (2014). Restoration of E-cadherin expression by selective Cox-2 inhibition and the clinical relevance of the epithelial-to-mesenchymal transition in head and neck squamous cell carcinoma. Journal of Experimental & Clinical Cancer Research.

[15] Qiu X, Brown KV, Moran Y, Chen D (2010). Sirtuin regulation in calorie restriction. Biochimica et Biophysica Acta.

[16] Cen Y, Youn DY, Sauve AA (2011). Advances in characterization of human sirtuin isoforms: chemistries, targets and therapeutic applications. Current Medicinal Chemistry.

[17] Oberdoerffer P, Michan S, McVay M, Mostoslavsky R, Vann J, Park SK, Hartlerode A, Stegmuller J, Hafner A, Loerch P, Wright SM, Mills KD, Bonni A, Yankner BA, Scully R, Prolla TA, Alt FW, Sinclair DA (2008). SIRT1 redistribution on chromatin promotes genomic stability but alters gene expression during aging. Cell.

[18] Michan S, Sinclair D (2007). Sirtuins in mammals: insights into their biological function. The Biochemical Journal.

[19] Webster BR, Lu Z, Sack MN, Scott I (2012). The role of sirtuins in modulating redox stressors. Free Radical Biology & Medicine.

[20] Dohadwala M, Yang SC, Luo J, Sharma S, Batra RK, Huang M, Lin Y, Goodglick L, Krysan K, Fishbein MC, Hong L, Lai C, Cameron RB, Gemmill RM, Drabkin HA, Dubinett SM (2006). Cyclooxygenase-2-dependent regulation of E-cadherin: prostaglandin E (2) induces transcriptional repressors ZEB1 and snail in non-small cell lung cancer. Cancer Research.

[21] Bozzo F, Bassignana A, Lazzarato L, Boschi D, Gasco A, Bocca C, Miglietta A (2009). Novel nitro-oxy derivatives of celecoxib for the regulation of colon cancer cell growth. Chemico-biological Interactions.

[22] Sitarz R, Leguit RJ, de Leng WW, Morsink FH, Polkowski WP, Maciejewski R, Offerhaus GJ, Milne AN (2009). Cyclooxygenase-2 mediated regulation of E-cadherin occurs in conventional but not early-onset gastric cancer cell lines. Cellular Oncology.

[23] Adhim Z, Matsuoka T, Bito T, Shigemura K, Lee KM, Kawabata M, Fujisawa M, Nibu K, Shirakawa T (2011). In vitro and in vivo inhibitory effect of three Cox-2 inhibitors and epithelial-to-mesenchymal transition in human bladder cancer cell lines. British Journal of Cancer.

[24] Wang H, Zhang H, Tang L, Chen H, Wu C, Zhao M, Yang Y, Chen X, Liu G (2013). Resveratrol inhibits TGF-β1-induced epithelial-to-mesenchymal transition and suppresses lung cancer invasion and metastasis. Toxicology.

[25] Cai Z, Wang Q, Zhou Y Zheng L, Chiu JF, He QY (2010). Epidermal growth factor-induced epithelial-mesenchymal transition in human esophageal carcinoma cells-a model for the study of metastasis. Cancer Letters.

[26] Wang J, Chen L, Li Y, Guan XY (2011). Overexpression of cathepsin Z contributes to tumor metastasis by inducing epithelial-mesenchymal transition in hepatocellular carcinoma. PLoS One.

[27] Kong C, Wang C, Wang L, Ma M, Niu C, Sun X, Du J, Dong Z, Zhu S, Lu J, Huang B (2011). NEDD9 is a positive regulator of epithelial-mesenchymal transition and promotes invasion in aggressive breast cancer. PLoS One.

[28] Gallo O, Franchi A, Magnelli L, Sardi I, Vannacci A, Boddi V, Chiarugi V, Masini E (2011). Cyclooxygenase-2 pathway correlates with VEGF expression in head and neck cancer. Implications for tumor angiogenesis and metastasis. Neoplasia.

[29] Kinugasa Y, Hatori M, Ito H, Kurihara Y, Ito D, Nagumo M (2004). Inhibition of cyclooxygenase-2 suppresses invasiveness of oral squamous cell carcinoma cell lines via down-regulation of matrix metalloproteinase-2 and CD44. Clinical & Experimental Metastasis.

[30] Kurihara Y, Hatori M, Ando Y, Ito D, Toyoshima T, Tanaka M, Shintani S (2009). Inhibition of cyclooxygenase-2 suppresses the invasiveness of oral squamous cell carcinoma cell lines via down-regulation of matrix metalloproteinase-2 production and activation. Clinical & Experimental Metastasis.

[31] Kim YY, Lee EJ, Kim YK, Kim SM, Park JY, Myoung H, Kim MJ (2010). Anti-cancer effects of celecoxib in head and neck carcinoma. Molecules and Cells.

[32] Ko SH, Choi GJ, Lee JH, Han YA, Lim SJ, Kim SH (2008). Differential effects of selective cyclooxygenase-2 inhibitors in inhibiting proliferation and induction of apoptosis in oral squamous cell carcinoma. Oncology Reports.

[33] Kim YS, Seol CH, Jung JW, Oh SJ, Hwang KE, Kim HJ, Jeong ET, Kim HR (2015). Synergistic effect of sulindac and simvastatin on apoptosis in lung cancer A549 cells through AKT-dependent downregulation of survivin. Cancer Research and Treatment.

[34] Bordone L, Guarente L (2005). Calorie restriction, SIRT1 and metabolism: understanding longevity. Nature reviews. Molecular Cell Biology.

[35] Giannakou ME, Partridge L (2004). The interaction between FOXO and SIRT1: tipping the balance towards survival. Trends in Cell Biology.

[36] Liu T, Liu PY, Marshall GM (2009). The critical role of the class III histone deacetylase SIRT1 in cancer. Cancer Research.

[37] Yamamoto H, Schoonjans K, Auwerx J (2007). Sirtuin functions in health and disease. Molecular Endocrinology.

[38] Byles V, Zhu L, Lovaas JD, Chmilewski LK, Wang J, Faller DV, Dai Y (2012). SIRT1 induces EMT by cooperating with EMT transcription factors and enhances prostate cancer cell migration and metastasis. Oncogene.

[39] Li L, ang L, Li L, Wang Z, Ho Y, McDonald T, Holyoake TL, Chen W, Bhatia R (2012). Activation of p53 by SIRT1 inhibition enhances elimination of CML leukemia stem cells in combination with imatinib. Cancer Cell.

[40] Pruitt K, Zinn RL, Ohm JE, McGarvey KM, Kang SH, Watkins DN, Herman JG, Baylin SB (2006). Inhibition of SIRT1 reactivates silenced cancer genes without loss of promoter DNA hypermethylation. Plos Genetics.

[41] Huffman DM, Grizzle WE, Bamman MM, Kim JS, Eltoum IA, Elgavish A, Nagy TR (2007). SIRT1 is significantly elevated in mouse and human prostate cancer. Cancer Research.

[42] Kwon HS, Ott M (2008). The ups and downs of SIRT1. Trends in Biochemical Sciences.

[43] Cha EJ, Noh SJ, Kwon KS, Kim CY, Park BH, Park HS, Lee H, Chung MJ, Kang MJ, Lee DG, Moon WS, Jang KY (2009). Expression of DBC1 and SIRT1 is associated with poor prognosis of gastric carcinoma. Clinical Cancer Research.

[44] Lee H, im KR, Noh SJ, Park HS, Kwon KS, Park BH, Jung SH, Youn HJ, Lee BK, Chung MJ, Koh DH, Moon WS, Jang KY (2011). Expression of DBC1 and SIRT1 is associated with poor prognosis for breast carcinoma. Human Pathology.

[45] Tseng RC, Lee CC, Hsu HS, Tzao C, Wang YC (2009). Distinct HIC1-SIRT1-p53 loop deregulation in lung squamous carcinoma and adenocarcinoma patients. Neoplasia.

[46] Suzuki K, Hayashi R, Ichikawa T, Imanishi S, Yamada T, Inomata M, Miwa T, Matsui S, Usui I, Urakaze M, Matsuya Y, Ogawa H, Sakurai H, Saiki I, Tobe K (2012). SRT1720, a SIRT1 activator, promotes tumor cell migration, and lung metastasis of breast cancer in mice. Oncology Reports.

[47] Firestein R, lander G, Michan S, Oberdoerffer P, Ogino S, Campbell J, Bhimavarapu A, Luikenhuis S, de Cabo R, Fuchs C, Hahn WC, Guarente L, Sinclair DA (2008). The SIRT1 deacetylase suppresses intestinal tumorigenesis and colon cancer growth. PLoS One.

[48] Sun L, Li H, Chen J, Dehennaut V, Zhao Y, Yang Y, Iwasaki Y, Kahn-Perles B, Leprince D, Chen Q, Shen A, Xu Y (2013). A SUMOylation-dependent pathway regulates SIRT1 transcription and lung cancer metastasis. Journal of the National Cancer Institute.

[49] Davies NM, McLachlan AJ, Day RO, Williams KM (2000). Clinical Pharmacokinetics and pharmacodynamics of celecoxib: a selective cyclo-oxygenase-2 inhibitor. Clin Pharmacokinet.

[50] Arico S, Pattingre S, Bauvy C, Gane P, Barbat A, Codogno P, Ogier-Denis E (2002). Celecoxib induces apoptosis by inhibiting 3-phophoinositide-dependent protein kinase-1 activity in the human colon cancer HT-29 cell line. J Biol Chem.

[51] Kim YS, Seol CH, Jung JW, Oh SJ, Hwang KE, Kim HJ, Jeong ET, Kim HR (2015). Synergistic effect of sulindac and simvastatin on apoptosis in lung cancer A549 cells through AKT-dependent downregulation of surviving. Cancer Res Treat.

[52] Saxena NK, Taliaferro-Smith L, Knight BB, Merlin D, Anania FA, O’Regan RM, Sharma D (2008). Bidirectional crosstalk between leptin and insulin-like growth factor-I signaling promotes invasion and migration of breast cancer cells via transactivation of epidermal growth factor receptor. Cancer Research.

[53] Keese CR, Bhawe K, Wegener J, Giaever I (2002). Real-time impedance assay to follow the invasive activities of metastatic cells in culture. BioTechniques.

