# Supplementary material for: Celecoxib and sulindac inhibit TGF-β1-induced epithelial-mesenchymal transition and suppress lung cancer migration and invasion via downregulation of sirtuin 1
Source: Oncotarget. 2016 Aug 9;7(35):57213–27. doi: 10.18632/oncotarget.11127 (PMC5302984; doi:10.18632/oncotarget.11127)
Supplement: Supplementary file 1 [file oncotarget-07-57213-s001.pdf]

Celecoxib and sulindac inhibit TGF-β1-induced epithelial-mesenchymal transition and suppress lung cancer migration and invasion via downregulation of sirtuin 1

SUPPLEMENTARY FIGURES

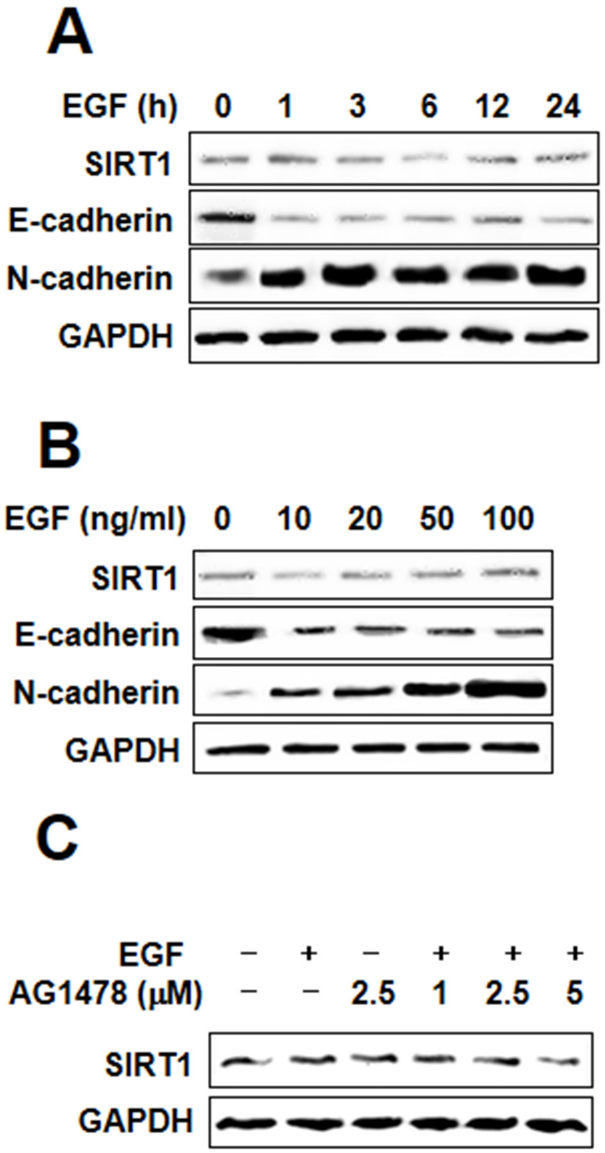

Supplementary Figure S1: Epidermal growth factor (EGF)-induced SIRT1 expression. A. and **B.** A549 cells were treated with EGF, and the expression of E-cadherin, N-cadherin, and SIRT1 was determined by immunoblotting. **C.** A549 cells were treated with 50 ng/mL EGF, with or without the indicated concentrations of AG1478 for 24h, and SIRT1 protein levels were examined by immunoblotting.

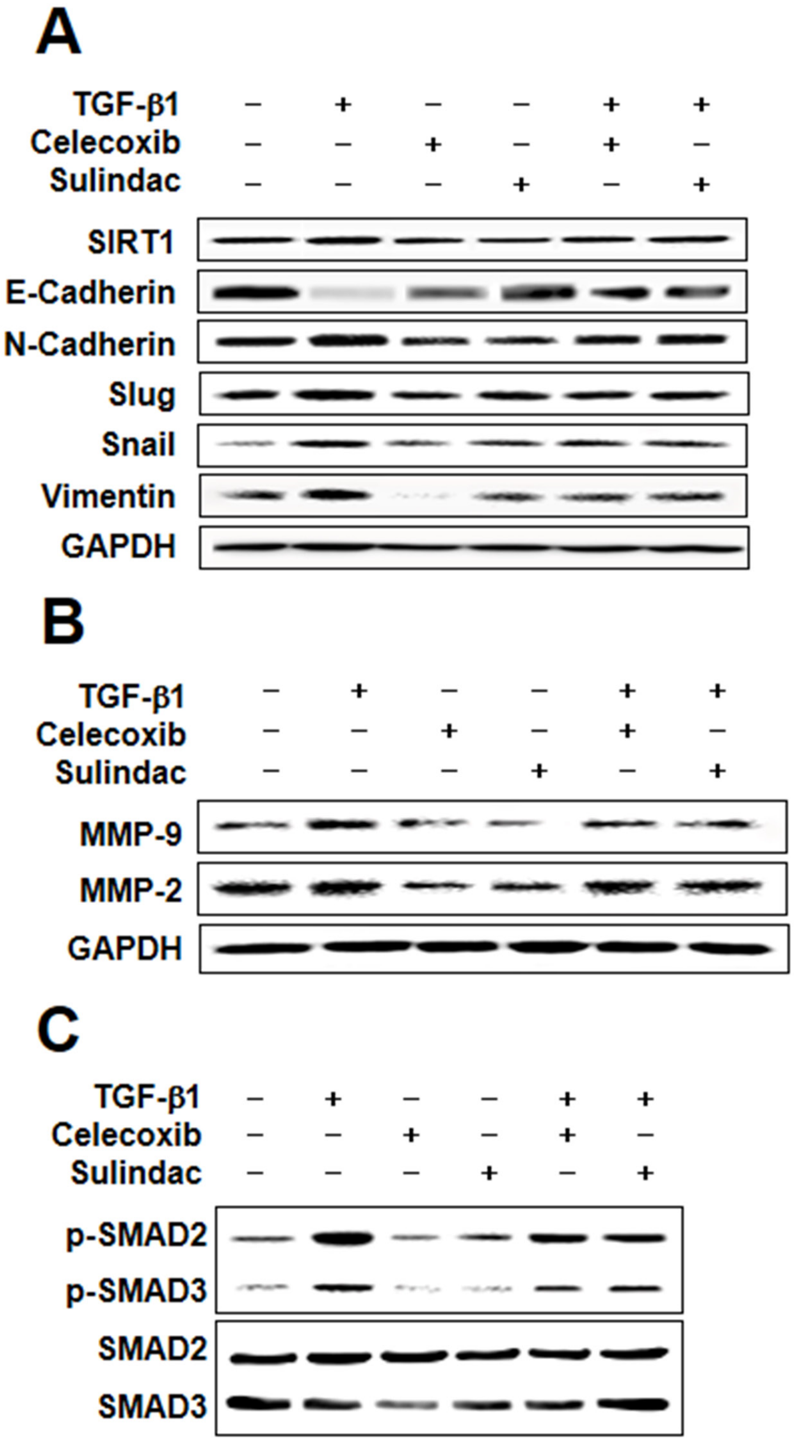

Supplementary Figure S2: Celecoxib and sulindac inhibit transforming growth factor (TGF)-β1-induced epithelial-mesenchymal transition in H460 cells. A. Western blot analysis using specific antibodies was performed to examine protein expression in whole cell lysates. **B.** H460 cells were stimulated with 5 ng/mL TGF-β1 for 2 h and then incubated with 10 µM celecoxib or 500 µM sulindac for 48 h. The cell lysates was subjected to 10% sodium dodecyl sulfate-polyacrylamide gel electrophoresis to measure the expression of MMP2 and MMP9. **C.** Cells were treated with 10 µM celecoxib or 500 µM sulindac in the absence or presence of 5 ng/mL TGF-β1. Cell lysates were then prepared and subjected to immunoblot analysis with antibodies to phosphorylated (p) or total forms of smad 2/3.

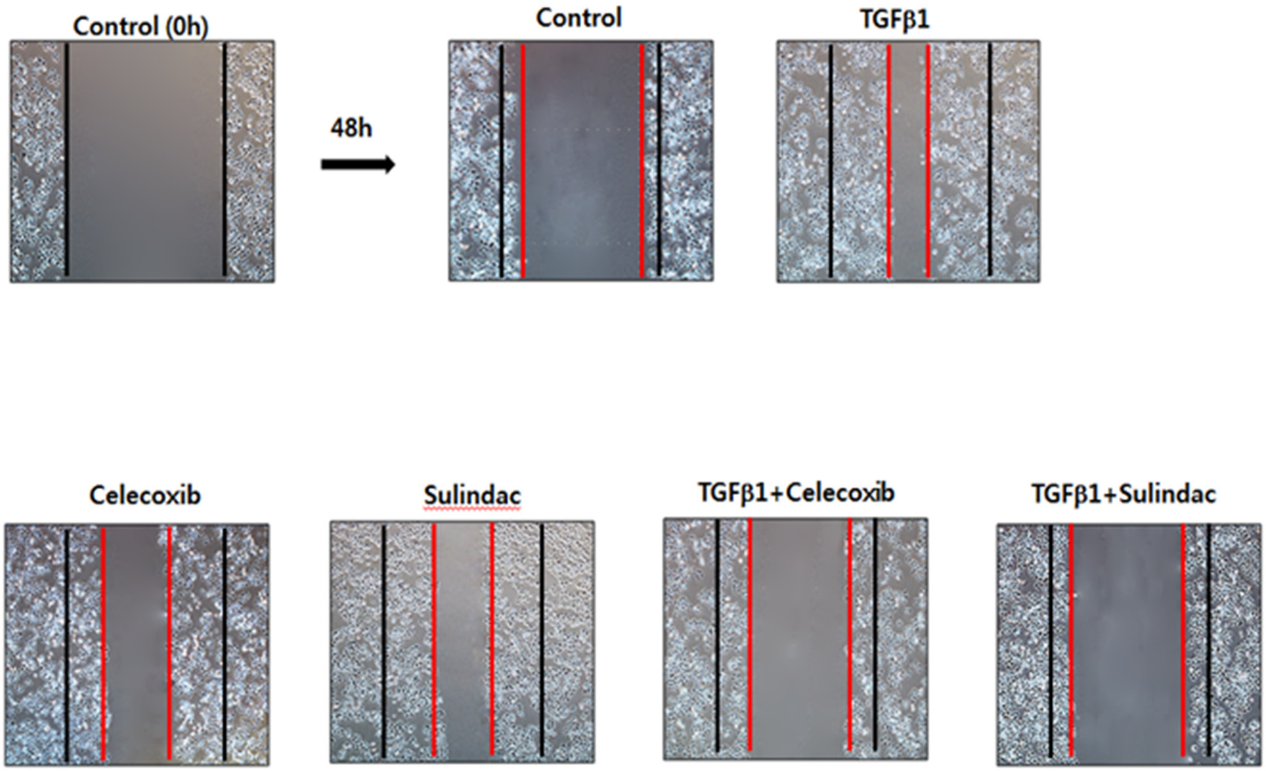

Supplementary Figure S3: Effects of celecoxib and sulindac on transforming growth factor (TGF)-β1-induced H460 cell migration. Cell migration was evaluated by wound healing assay. The confluent H460 monolayer was scratched with a pipette tip and washed to remove the debris. Fresh medium containing 0.5% serum was then added. Red lines indicate the cell edges at the T_0 point.

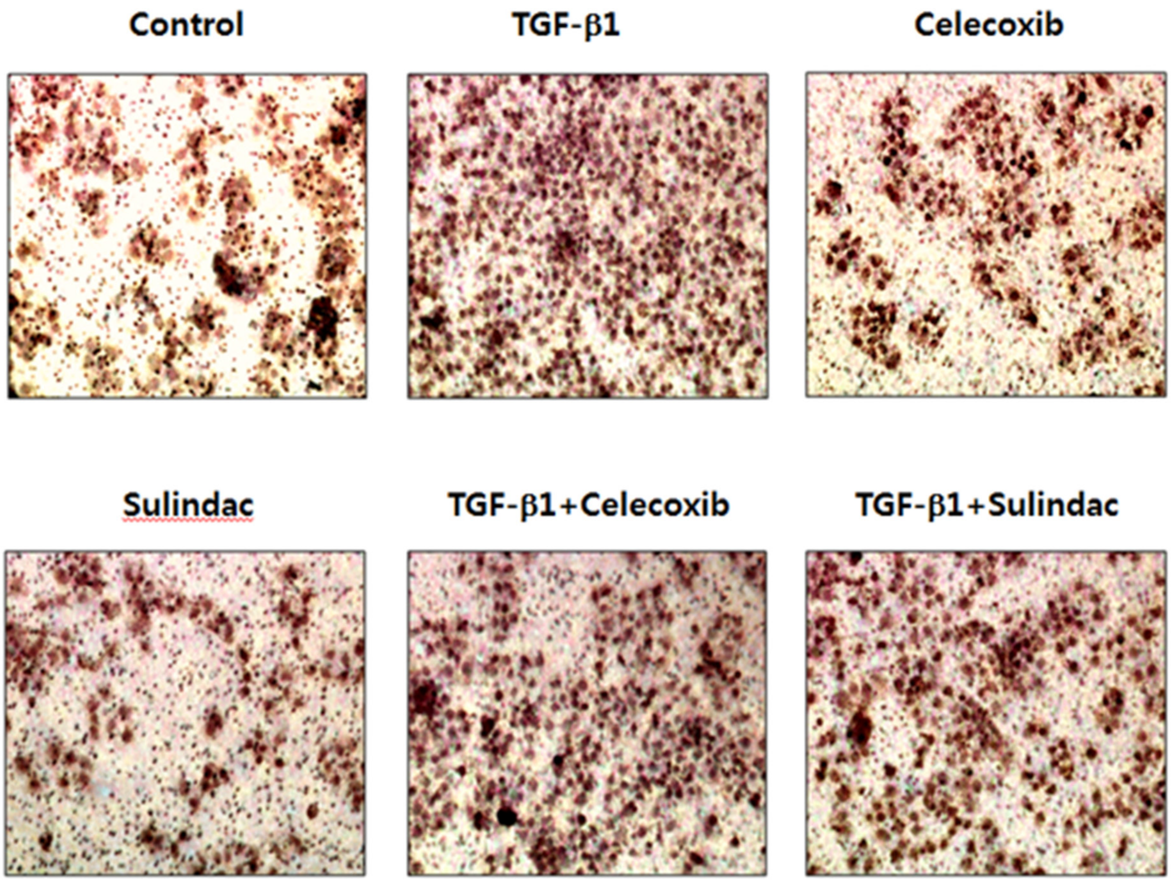

Supplementary Figure S4: Effects of celecoxib and sulindac on transforming growth factor (TGF)-β1-induced H460 cell invasion. Effect of celecoxib and sulindac on H460 cell invasion in a 200× light microscope after crystal violet staining by matrigel invasion assay as described in Materials and Methods.

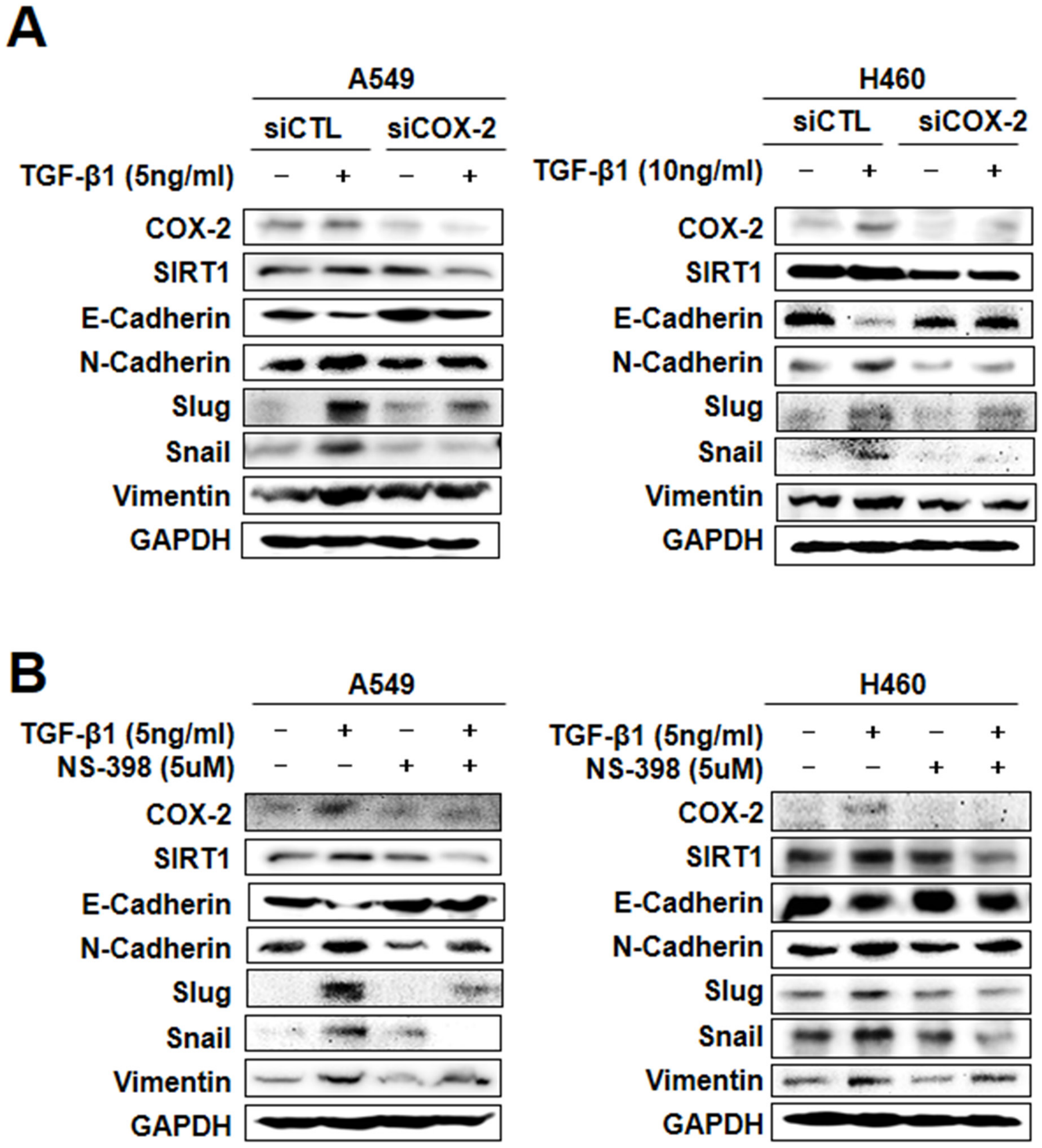

Supplementary Figure S5: Effect of COX-2 in transforming growth factor (TGF)-β1-induced epithelial-mesenchymal transition (EMT) inhibited by celecoxib and sulindac. A. and **B.** Cells were transfected with COX-2 siRNA or treated with the COX-2 inhibitor NS-398, and then further incubated in the presence of celecoxib or sulindac for 24 h. The cell lysates of each group were prepared and probed for SIRT1 and EMT hallmarks by western blot.
